# Detection of Biochemical Pathways by Probabilistic Matching of Phyletic Vectors

**DOI:** 10.1371/journal.pone.0005326

**Published:** 2009-04-24

**Authors:** Hua Li, David M. Kristensen, Michael K. Coleman, Arcady Mushegian

**Affiliations:** 1 Stowers Institute for Medical Research, Kansas City, Missouri, United States of America; 2 Department of Microbiology, Molecular Genetics, and Immunology, University of Kansas Medical Center, Kansas City, Kansas, United States of America; Utrecht University, Netherlands

## Abstract

A phyletic vector, also known as a phyletic (or phylogenetic) pattern, is a binary representation of the presences and absences of orthologous genes in different genomes. Joint occurrence of two or more genes in many genomes results in closely similar binary vectors representing these genes, and this similarity between gene vectors may be used as a measure of functional association between genes. Better understanding of quantitative properties of gene co-occurrences is needed for systematic studies of gene function and evolution. We used the probabilistic iterative algorithm Psi-square to find groups of similar phyletic vectors. An extended Psi-square algorithm, in which pseudocounts are implemented, shows better sensitivity in identifying proteins with known functional links than our earlier hierarchical clustering approach. At the same time, the specificity of inferring functional associations between genes in prokaryotic genomes is strongly dependent on the pathway: phyletic vectors of the genes involved in energy metabolism and in *de novo* biosynthesis of the essential precursors tend to be lumped together, whereas cellular modules involved in secretion, motility, assembly of cell surfaces, biosynthesis of some coenzymes, and utilization of secondary carbon sources tend to be identified with much greater specificity. It appears that the network of gene coinheritance in prokaryotes contains a giant connected component that encompasses most biosynthetic subsystems, along with a series of more independent modules involved in cell interaction with the environment.

## Introduction

Phyletic vectors were first introduced by Tatusov et al. [1] in their work on the Clusters of Orthologous Groups (COGs). A *phyletic vector* of a COG or of any gene indicates the binary presence or absence of orthologs of this COG or gene in a series of completely sequenced genomes. Each “measurement”, or vector coordinate, is assigned as a “1” if there is at least one ortholog contained in a genome and a “0” if not. The authors pointed out that phyletic vectors (which they called phylogenetic patterns) were different for different functional classes of proteins/COGs. It is reasonable to assume that closely related phyletic vectors (essentially the same construct is also known as phylogenetic profile [2]) suggest co-inheritance of these genes in the evolutionary history of the involved organisms, and thus could be used to infer possible functional linkages between genes/proteins. There have been many studies to quantitatively assess the utility of phyletic vectors for predicting such linkages. Various distance measures between phyletic vectors have been developed and evaluated, including correlation-based distance, mutual information, a “trait-to-gene” matching based on set theory, “phenotype propensity”, etc. [3]–[9]. The lists of new predictions accompanied each publication, some of them were confirmed experimentally, and many more are waiting to be tested. Interactive web servers have also been set up to allow exploration of gene co-inheritance [10].

Despite all this interest and the successes of phyletic vectors in prediction of protein function, the quantitative properties of phyletic vectors, and of distances between them, remain to be studied in detail. One of the most important questions here concerns the proper way to measure distances/similarities between high-dimensional vectors. Speaking formally, different distance measures vary in their quantitative behavior, and this may impact the sensitivity and specificity of vector comparison. From the biological point of view, it is quite clear that even those genes that participate in the same pathway may not be co-inherited in perfect synchrony, for a multitude of reasons, including complex topology of metabolic networks that results in redundancy of some gene products and polyfunctionality of others [11]; non-orthologous displacement of isofunctional genes [12]; and the trend of profuse gene loss in the genomes of parasitic microorganisms (which account for a substantial fraction of all sequenced genomes) and even in the free-living microbes with large genomes [13]. Thus, it is important to devise ways to find groups of genes that are more likely to be gained or lost together than random, even if those gains and losses do not occur in a complete lockstep. In fact the main, if sometimes implicit, goal of all described methods of phyletic vector analysis has been to find the best way of treating the imperfect matches between vectors.

Recently, the problem of comparison of gene vectors was studied in the framework of explicit probabilistic pattern matching, inspired by popular programs for sequence database searches, such as PSI-BLAST [14], and was implemented in the program called Psi-square [15]. Tests on three different types of gene vectors (phyletic vectors, gene expression vectors and protein-protein interaction vectors) indicate that Psi-square usually is more sensitive and sometimes also more specific than other methods, including those that have been devised specifically for analysis of each type of genome-wide vector space [15].

Psi-square algorithm allows us to obtain the list of nearest database neighbors for each vector. In this work, we used an extended Psi-square algorithm that incorporates pseudocounts to analyze the space of phyletic vectors further. Our results indicate that the extended algorithm yields more complete descriptions of functional pathways than the original version, and thus helps to predict novel protein functions and perhaps to understand their evolution better.

## Results

### Phyletic vector matching using a Psi-square program: a version that uses pseudocounts

We have a dataset represented by an m by 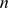 matrix and the binary (i.e., presence/absence, or 1/0) data in each cell: the *i* ^th^ row is called the *i* ^th^ vector and we have 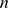 data points associated with each row (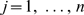). We want to find vectors in the database (the whole dataset) that are most similar to the query vector, which we do according to the following steps (see [15] for more detail):

1. For a given query vector, first quickly scan the dataset to identify a set of vectors that are highly similar to the query at the given threshold *r*.
2. Construct a *profile* from this set of vectors (“target set”) in the form of a dimension-specific scoring matrix (DSSM):
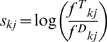


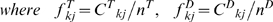
for 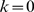, 1 (the presence/absence data), 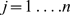, where 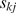 is the score of value 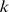 at vector coordinate 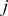, 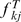 is the probability of value 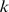 at the 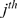 column in the target set and 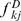 is the probability of 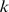 at the 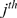 column in the database, 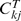 and 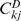 are counts of value k's in the target set and the database, respectively; and 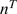 and 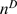 are the number of vectors in the target set and the database.
3. Calculate similarity scores for every vector in the database based on the DSSM as: 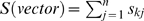. Select vectors with high scores based on a threshold (S).
4. Add selected vectors to the target vector set.
5. Repeat step 2 until no new vectors can be matched.

**Figure 1:**
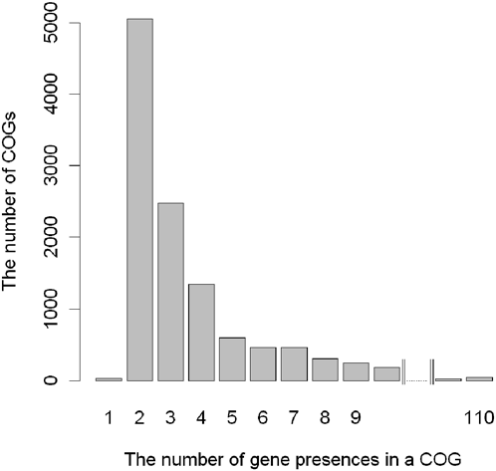
The distribution of the COG-06 dataset by the number of genomes that encode a representative of this COG (i.e., by the number of coordinates set at 1 in the phyletic vector corresponding to each COG). Only 1,021 proteins (7%) are encoded in more than half of the 110 genomes.

In many types of genome-scale data, vector coordinates are dominated by zeroes, because most genes do not produce a signal under most conditions in a given measurement space. In the current work, this corresponds to the observation that most genes are found in a minority of genomes. The COG dataset that we used (COG-06 dataset) includes 14,714 phyletic vectors (COGs) with 110 coordinates (genomes), and in this dataset 88.7% of all vector coordinates have the value of zero, about half of vectors have three or less of their coordinates equal to one, and only 7% of vectors have more than half of their coordinates equal to one (Figure 1). In this situation, gene presence typically enters a probabilistic model with the frequency of zero, 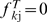. When calculating 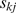 for the DSSM, the initial Psi-square algorithm assigned 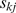 as 0, which means that this coordinate does not contribute to the score of a new vector. In this work, with the goal to retrieve all functional associations for a given query vector, we have entered a pseudocount, a background frequency calculated from the complete dataset, into the calculation of 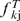 as follows:
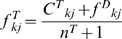



In this case, 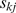 for the non-zero count of 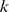 (C^T^_kj_) has only a negligible effect on the DSSM; however, for the zero count of 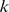,
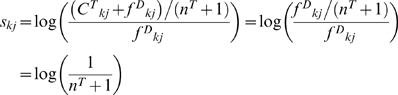
and thus 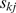 is always a negative score, rather than 0 as in the original version. During the iterative search, the vectors not matching to the profile get relatively lower scores so that we could identify more closed related vectors.

### Pseudocounts increase sensitivity

To evaluate the impact of pseudocounts on Psi-square performance, we used 52 pathways and functional systems and the COG-06 dataset, representing 6,651 distinct phyletic vectors (the list of pathways is given in Table S1). We studied the effect of pseudocounts on the sensitivity of detection of these known pathways. To initiate the Psi-square algorithm, we chose one or more representative vectors from each of 52 pathways and functional systems to be the query (one vector for pathways with relatively uniform phyletic patterns and two or three vectors for pathways with relatively diverse patterns). Typically, the complement of the correlation coefficient was used as the distance measure when constructing a DSSM during the first iteration and the threshold varied from 0.6 to 0.8 (see Methods section for notes on selection of the appropriate distance measure and threshold). If more then one query was used for a particular pathway, the results of the searches for all queries were merged. The sensitivity of these searches, defined as the percentage of recovered COGs that belong to the same pathway or functional system as the query, was measured for each of the 52 pathways and averaged.

**Figure 2:**
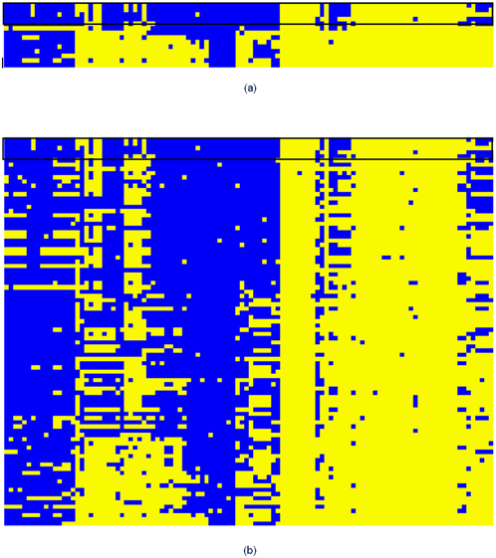
Phyletic patterns of COGs recovered in Psi-square searches (a) without pseudocounts and (b) with pseudocounts. In both, the same query COG01905 (the 24 kD subunit of NADH ubiquinone oxidoreductase complex) and the same parameters were used to search the target COG-06 database. The x axis represents genomes and the y axis represents COGs (See Table S7 for the detailed lists). The boxed part includes the patterns of five COGs in the initial DSSM.

The use of pseudocounts improved the average sensitivity from 64.7% to 71.6%. For example, the NADH-ubiquinone oxidoreductase complex (Complex I) in most bacteria consists of 14 core subunits [16], but some bacteria and most archaea lack several of them. When COG01905 (the 24 kD subunit) was used as the query, we recovered 13 of the total 15 known Complex I components, compared to only 4 relevant COGs recovered without pseudocounts (Figure 2).

In another example, we compared phyletic vectors of the machinery involved in assembly and function of bacterial flagella. In the best-studied case of *Salmonella typhimurium*, there are more than 40 components involved in various aspects of flagella function, but only about 24 of them are broadly conserved in other motile bacteria and are functionally indispensable [17]. These genes map to 21 COGs. We asked whether some of the more variable components of flagella could be used as queries that would specifically recover these core genes. We selected COG01298 (FlhA), COG02882 (Flagellar biosynthesis chaperone), COG01317 (FliH), COG01334 (FlaG), and COG02747 (anti-sigma28 factor) as query vectors and performed Psi-square searches with pseudocounts, again using the correlation coefficient as the initial distance measure and setting the threshold around 0.7. For each of these five COGs, Psi-square was able to recover all 21 COGs, and additionally found between 64 and 167 lower-scoring genes including 11–15 genes from the variable subset of flagellar proteins (Table 1). On average, the use of pseudocounts increases the sensitivity by about 20% compared to the original algorithm in this example.

**Table 1:** Comparison of Psi-sqaure with and without pseudocounts in numbers of true positive (TP) and sensitivity.

COG	Definition	With pseudocount		Without pseudocount	
		TP	Sensitivity	TP	Sensitivity
01298	Flagellar biosynthesis pathway; component FlhA	32	0.78	26	0.63
01317	Flagellar biosynthesis/type III secretory pathway protein	34	0.83	26	0.63
01334	Uncharacterized flagellar protein FlaG	36	0.88	24	0.59
02747	Negative regulator of flagellin synthesis	35	0.85	26	0.63
02882	Flagellar biosynthesis chaperone	32	0.78	25	0.61

The table shows Psi-sqaure search results for the tightly linked group of proteins involved in flagellum structure and biogenesis. Based on our current knowledge, 41 COGs (TP) participate in flagellum structure and biogenesis pathway, including 29 known COGs defined in the pathway, 8 chemotaxis related COGs, and 4 experimentally verified proteins (COG02747, COG03144, COG03190, and COG04787).

These examples indicate that probabilistic modeling of phyletic vectors is a sensitive way of finding groups of related vectors. On a related note, probabilistic approaches may help to overcome “the curse of non-transitivity”, where sensitivity of a database search may be biased by the choice of the query vector. As is often the case with the Psi-square algorithm, an analogy can be observed in sequence searches, where increasingly sophisticated probabilistic models of sequence families are used to establish links between sequences, but outlying members of even well-studied families continue to be discovered [18].

### Growth of the COG database and probabilistic search strategy separately contribute to improved sensitivity of pathway discovery

We next compared the performance of the extended version of the Psi-square algorithm with our earlier results on pathway discovery [3], which employed hierarchical clustering of phyletic vectors. Here, we asked two main questions: first, whether probabilistic iterative search of the database of phyletic vectors results in a significant, across-the-board improvement in discovery of functionally linked proteins as compared to a more rigid hierarchical clustering approach; and second, whether this discovery rate is further improved by including more species and more proteins into the analysis.

We first used the COG-04 dataset (see Methods) to compare the the extended Psi-square algorithm with our hierarchical clustering approach that used the same data [15]. Our results indicate that the iterative probabilistic Psi-square algorithm gives better pathway coverage than hierarchical clustering of the same dataset, i.e., 66% compared to 50% (p-value <0.01 using Welch two-sample t-test). Improvements in sensitivity were seen in 34 out of 52 pathways, in particular with those pathways that include phyletic vectors not closely similar to each other, i.e., those pathways whose evolutionary history may have been richer in asynchronous gene gains and losses (Table S2). For example, hierarchical clustering of phyletic vectors has indicated that a subset of VirB genes, including VirB4 and VirB8-11, represented a discrete module disjoint from the rest of VirB genes [3]. Using VirB3 (COG03702) as the query vector, we recovered 110 COGs with 2 iterations; when sorted by the distance from the query, the first 15 matches contained VirB6 and VirB8-10 (VirB11 is a large COG encumbered by paralogs, and is found only at the bottom of the ranked list). Thus, Psi-square identified the link between two subsystems of T4SS, again resulting in higher sensitivity than earlier approaches.

COG-06 contains almost twice as many microbial genomes as COG-04, including representatives of several additional diverse clades of prokaryotes, such as Delta-proteobacteria, Bacillus, and Corynebacterium. Additional members have been added in most of the existing clades as well, including 15 more genomes in Gramma-proteobacteria, three more in Epsilon-proteobacteria, etc. Furthermore, the number of COGs in COG-06 is almost three times as high as in COG-04. Here we asked whether these increases were beneficial for our goal of discovering functional links between genes, using the same benchmark of 52 known pathways and functional systems. The sensitivity of Psi-square analysis with the COG-06 database was 71.6% on average, compared to 66% for COG-04, indicating that the larger dimensionality of the phyletic vectors and perhaps their higher complexity as well, provides better identification of co-inherited groups of genes using phyletic patterns (Table S2). In contrast, the original Psi-square algorithm shows little difference in sensitivity between the COG-06 and COG-04 datasets, indicating that pseudocounts help best when the dataset contains more sparse data.

### Two classes of pathways on which the specificity of Psi-square is sharply different

The main conclusion from our analysis thus far is that a probabilistic approach to phyletic vector matching provides commendable sensitivity across a wide range of functions in finding the co-inherited members of the same cellular pathway. The specificity of the method, however, is more complicated to assess, since there is a dramatic difference in the ability of Psi-square to recover different cellular pathways and subsystems. In particular, we have found that a large portion of the central cellular metabolism consists of multiple pathways represented by genes with strongly similar phyletic vectors, so that a probabilistic model of one pathway was not able to distinguish between this pathway and other subsystems. At the same time, there were other pathways which can be represented by much more specific DSSMs. In this section, we describe this distinction in more detail, using the pathways for de novo biosynthesis of nucleotides and of amino acids as an example of the former trend, and several subsystems of secondary metabolism and cell envelope assembly as examples of the latter.

Most of the free-living bacteria and archaea encode all enzymes in nucleotide biosynthesis, whereas parasitic microorganisms with small genomes typically retain only a small subset of these genes, often restricted to the base salvage and thymidylate synthesis. Similarity searches group together the proteins in the purine biosynthesis pathway and pyrimidine biosynthesis pathway, making it difficult to distinguish between these two pathways based on phyletic patterns alone. More specifically, 18 COGs are involved in purine biosynthesis and 14 COGs in pyrimidine biosynthesis. Using COG00138 from the former and COG00044 from the latter group as query vectors, we found, respectively 256 and 214 COGs, with significant overlaps (156 COGs) between them. Among the 256 COGs found by the purine biosynthesis query, seven COGs that belong to the pyrimidine biosynthesis are found in the first iteration. Some of these seven COGs are much more closely related to the query COG00138 than other COGs in the purine biosynthesis pathway. Similarly, 13 COGs that belong to the purine biosynthesis pathway were retrieved by COG00044 before convergence, without clear separation from the pyrimidine biosynthesis genes. Searches with other queries in these pathways provide similar results. This suggests that in these searches, the enzymes for biosynthesis of purines and those of pyrimidines start to intermingle with each other at an early iteration and the pathway models tend to become indistinguishable in the later iterations of Psi-square search.

The situation with amino acid biosynthesis is similar. Biosynthesis of most amino acids can be seen as a hierarchy, in which a group of related pathways is associated with a specific precursor, often derived from the citric acid cycle or glycolysis intermediates [19]. Because of the shared biosynthetic enzymes in the trunk portion of each of these groups of pathways, it does not come as a surprise that a COG involved in biosynthesis of one amino acid, when used as a Psi-square query, often retrieves components of other pathways in the same group. Perhaps less expected is the high similarity of the phyletic vectors that belong to different families of amino acid biosynthesis pathways. For example, using COG00141 (one of 12 COGs in the histidine biosynthesis pathway) as a query, we retrieved 9 other COGs in the same pathway, but also 8 out of 10 COGs from the Ile/Leu/Val pathway, and 16 COGs involved in biosynthesis of other amino acids. Thus, similarly to the case of purine and pyrimidine biosynthesis pathways, genes involved in amino acid biosynthesis appear to share phyletic vectors to such an extent that it is difficult to specifically distinguish individual amino acid biosynthesis pathways by comparing phyletic vectors alone.

In all these searches, applying conservative thresholds for model inclusion tended to cause abrupt decomposition of the pathways into small fragments, typically representing stoichiometric subunits of the enzymatic complexes (data not shown), in agreement with our earlier observations made using hierarchical clustering [3]. On the other hand, permissive thresholds that produce more sensitive probabilistic models and improve the recovery of the known components of a given pathway, typically also result in multiple matches from other pathways. Using an analogy from the analysis of complex networks, if genes are modeled as nodes and high similarity of two phyletic vectors represents an edge connecting two genes that are characterized by these vectors, then most pathways of the TCA cycle and of *de novo* biosynthesis of nucleotides and amino acids will probably form one giant, not strongly modular, connected component in the co-inheritance network.

We found, however, that the state of affairs is different in the pathways involved in complex coenzyme biosynthesis and in interactions of cells with their environment. These functional categories of genes are much more amenable to delineation using the probabilistic matching of their phyletic vectors. For example, cobalamin (vitamin B12) is a tetrapyrrole derivative that is used as a cofactor of many enzymes. Phyletic vectors of different enzymes of vitamin B12 biosynthesis are vastly different: for example, cobalamin-5-phosphate synthase CobS/CobV (COG00368) catalyzes the last step in the reaction and is found in 62 species out of 110 examined in this work, whereas precorrin-6× reductase CbiJ/CobK (COG02099) catalyzes an early step and is found in only 30 species. Nevertheless, when the latter is used as the query in Psi-square search using Simpson similarity index as the distance measure and 0.30 as the threshold for building the initial DSSM, it retrieves at the first step four members of the same pathway found in 27–42 species. When this group of vectors is used to build a DSSM and search the database again, it produces a list of 42 matches (Table 2). The top half of the ranked list contains 12 confirmed members of the pathway and six COGs without known connection to cobalamine metabolism, and the second half of the list includes three more factors of cobalamine metabolism, which nearly complete the pathway makeup. Notably, the last true positive in the list, cobalt permease (COG00310) is found in only 24 species and its phyletic vector is 38 bits apart from CobS/CobV, which was also found in the same iteration (Table 2). The inspection of the other COGs on the list identifies at least one candidate with a potential link to cobalamine function: COG03920, which encodes a signal-transducing histidine kinase. This COG has undergone lineage-specific expansions in several archaea and in bacteria of the order *Rhizobiales*. In contrast, in *Clostridium*, *Listeria*, and *Fusobacterium* COG03920 is not highly duplicated and is adjacent on the chromosome to the ethanolamine utilization operon, which encodes a cobalamine-dependent enzyme, ethanolamine-ammonia lyase. It is plausible that COG03920 in these species is co-inherited and perhaps co-regulated with the cobalamine biosynthesis genes, as a way to coordinate the expression of the enzyme and the accumulation of its cofactor.

**Table 2:** Psi-square search results using COG02099 as the query.

match_ID	cat	function	Iter.	distance	score
COG02099	H	Precorrin-6× reductase	0	0	−1
COG01429	H	Cobalamin biosynthesis protein CobN and related Mg-chelatases	0	0.267	−1
COG02073	H	Cobalamin biosynthesis protein CbiG	0	0.278	−1
COG02082	H	Precorrin isomerase	0	0.286	−1
COG01010	H	Precorrin-3B methylase	0	0.286	−1
COG02243	H	Precorrin-2 methylase	1	0.302	79.906
COG02875	H	Precorrin-4 methylase	1	0.318	77.972
COG02241	H	Precorrin-6B methylase 1	1	0.318	73.949
**COG03707**	**T**	**Response regulator with putative antiterminator output domain**	**1**	**0.333**	**0.65**
COG01903	H	Cobalamin biosynthesis protein CbiD	1	0.353	52.597
COG01797	H	Cobyrinic acid a;c-diamide synthase	1	0.356	71.256
COG02242	H	Precorrin-6B methylase 2	1	0.364	67.15
**COG01402**	**R**	**Uncharacterized protein; putative amidase**	**1**	**0.382**	**23.498**
COG02087	H	Adenosyl cobinamide kinase/adenosyl cobinamide phosphate guanylyltransferase	1	0.391	17.504
**COG03339**	**S**	**Uncharacterized conserved protein**	**1**	**0.4**	**2.996**
COG01492	H	Cobyric acid synthase	1	0.444	54.227
COG01270	H	Cobalamin biosynthesis protein CobD/CbiB	1	0.464	55.07
**COG02308**	**S**	**Uncharacterized conserved protein**	**1**	**0.467**	**0.543**
**COG03920**	**T**	**Signal transduction histidine kinase**	**1**	**0.467**	**1.009**
**COG01233**	**Q**	**Phytoene dehydrogenase and related proteins**	**1**	**0.474**	**6.508**
COG01240	H	Mg-chelatase subunit ChlD	1	0.5	11.571
COG00368	H	Cobalamin-5-phosphate synthase	1	0.516	43.335
COG02109	H	ATP:corrinoid adenosyltransferase	1	0.519	21.555
**COG02020**	**O**	**Putative protein-S-isoprenylcysteine methyltransferase**	**1**	**0.528**	**0.645**
**COG00145**	**E**	**N-methylhydantoinase A/acetone carboxylase; beta subunit**	**1**	**0.533**	**9.969**
**COG03387**	**G**	**Glucoamylase and related glycosyl hydrolases**	**1**	**0.533**	**1.061**
COG01239	H	Mg-chelatase subunit ChlI	1	0.533	8.586
**COG01082**	**G**	**Sugar phosphate isomerases/epimerases**	**1**	**0.536**	**11.341**
COG02038	H	NaMN:DMB phosphoribosyltransferase	1	0.548	34.104
**COG01364**	**E**	**N-acetylglutamate synthase (N-acetylornithine aminotransferase)**	**1**	**0.554**	**5.419**
**COG01533**	**L**	**DNA repair photolyase**	**1**	**0.565**	**9.048**
COG00146	E	N-methylhydantoinase B/acetone carboxylase; alpha subunit	1	0.567	1.042
**COG00310**	**P**	**ABC-type Co2+ transport system; permease component**	**1**	**0.567**	**5.422**
COG01994	R	Zn-dependent proteases	1	0.568	0.408
COG00378	O	Ni2+-binding GTPase involved in regulation of expression and maturation of urease and hydrogenase	1	0.571	3.752
COG02202	T	FOG: PAS/PAC domain	1	0.574	2.619
COG01741	R	Pirin-related protein	1	0.577	0.398
COG01201	R	Lhr-like helicases	1	0.581	10.08
COG00467	T	RecA-superfamily ATPases implicated in signal transduction	1	0.581	10.199
COG01305	E	Transglutaminase-like enzymes; putative cysteine proteases	1	0.582	3.664
COG00182	J	Predicted translation initiation factor 2B subunit; eIF-2B alpha/beta/delta family	1	0.595	10.772
COG01878	R	Predicted metal-dependent hydrolase	1	0.595	4.143

We have ordered 52 pathways in the order of specificity with which they could be identified by Psi-square in this work. Although the absolute values of specificity are generally low (below 0.2 for most pathways), the trend exemplified above is clear: pathways of coenzyme biosynthesis and of cell surface component assembly have higher specificity than central pathways of intermediary metabolism.

### Discovering new components of poorly characterized pathways and molecular correlates of phenotypic traits

Many of our searches that were initiated with phyletic vectors of poorly characterized proteins resulted in identification of relatively compact groups of vectors, often containing a subgroup of functionally linked genes along with uncharacterized proteins. For example, a specialized Type VI secretion system (T6SS) has been recently described in gammaproteobacteria [20]–[21]. T6SS is made up of distinct molecular components, apparently not shared with other functionally similar systems such as Type II and Type IV secretion systems. The proteins that are thought to form the core of the T6SS correspond to COGs 03157, 03455, 03466, 03501, and 03515–03523. However, our analysis indicates that this group is coinherited together with many other genes, including COGs 02975, 03009, 03026, 03123, 03124, 03132, 03148, 03150, 03164, 03497, 03530, 04575, and 04681. We predict that many, if not all, of these genes are involved in T6SS. Interestingly, there are virtually no matches in Psi-square searches to T6SS and T4SS, and there is only a small degree of cross-matching to T3SS, each of which also operates in many gammaproteobacteria.

A distinct way to define a phyletic vector is to code the phenotypic traits of organisms as binary character states, and see whether matching vectors can be found in the database. This idea has been used to find genes whose phyletic vectors correlate with ecological factors, such as extremely high-temperature habitats [22], or with biochemical properties, such as the ability to incorporate selenocysteine into proteins [23]. In this case again, the match between phenotypic trait and gene presence is usually imperfect, primarily because of gene displacements and functional takeovers, or, in other words, relatively frequent functional convergence of genes at the molecular level, when different species use proteins with unrelated sequence and structure to perform a molecular function that results in the same phenotype. Psi-square allows us to match traits to genes by searching the COG dataset using phyletic vectors of specific traits as queries.

In order to find molecular correlates of different phenotypes, ecotypes, or disease symptoms associated with various prokaryotes, we derived 20 phenotypic vectors (Table S3) representing the presence or absence of a given phenotype in 110 genomes, without any requirement that a COG with a corresponding phyletic vector actually exists in the data. We then applied Psi-square to find groups of the co-inherited COGs similar to these phenotypes. The success of this approach was quite modest: in most cases there were no well-defined groups of genes with a clear connection to a known phenotype. This suggests that most strategies of biological survival and adaptation to various lifestyles may require many simultaneous changes at molecular and cellular levels, rather than facile gain and loss of discrete groups of genes. Nonetheless, several correlations were noted. In addition to the relatively well-studied case of prokaryotic cell motility, which strongly correlates with the presence of genes involved in flagella assembly and chemotaxis ([24], Table S4), we found that the phyletic vectors of reverse gyrase (COG01110), previously proposed to be a strict determinant of hyperthermophily [22], remains the closest match to the phenotypic vector of hyperthermophily, even though it is not found in moderately thermophilic bacteria (but note that a plasmid-encoded copy of an orthologous gene has been identified in bacterium *Thermus thermophilus* strain HB8 [25]). Interestingly, COG01756, a putative SPOUT-domain rRNA methyltransferase, appears to be equally close to the desired phenotype, but this COG01756 is distinct from reverse gyrase by omnipresence of the former, and complete absence of the latter, in eukaryotic genomes which were not included in COG-06.

The second observation of considerable interest concerns a strong correlation between the “food-poisoning” phenotype and the ethanolamine utilization genes. There are 11 genomes in our dataset that belong to bacteria associated with food poisoning. These bacteria are from two genera of Gram-positive bacteria (*Bacillus* and *Clostridium*) and two clades in Gram-negative *Proteobacteria* (*Escherichia*, *Salmonella* and *Bordetella* in gammaproteobacteria and *Listeria* in epsioloproteobacteria (Table S5). The closest match to this phenotype is represented by three COGs with the same phyletic vector: COG04810, COG04816, and COG04917. These COGs are present in 12 genomes, seven of which show a food-poisoning phenotype. All three COGs are annotated as ethanolamine utilization proteins, and the next two iterations of the Psi-square search detect, among many uncharacterized COGs, several additional COGs with assigned roles in ethanolamine utilization, as well as in biosynthesis of the cobalamine cofactor of ethanolamine lyase. The involvement of ethanolamine catabolism and/or of the paralogous system of propionate catabolism in pathogenicity of food has been suggested by Korbel et al. [26] on the basis of a data-mining approach that combined several lines of genomic evidence with parsing of Pubmed abstracts. Our results appear to primarily implicate three COGs, none of which, however, is directly responsible for enzymatic transformation of ethanolamine. COG04810 and COG04816 encode the structural subunits of an auxiliary organelle, the metabolosome, which is thought to compartmentalize ethanolamine utilization, and COG04917 is a transporter of the ABC class with unknown specificity. Thus, the toxic agent that causes food poisoning is likely to be associated with the metabolosome formation or function, but its molecular identity remains to be discovered.

## Discussion

Phyletic vectors of genes encoded by completely sequenced genomes are thought to be a valuable resource for predicting gene functions and identifying functional modules, but there is no standard approach to analysis of co-inherited groups of genes [27]. In this work, we applied a probabilistic approach to the problem of approximate matching of phyletic vectors. Our data indicate that probabilistic models of phyletic vectors, which can be updated in the course of iterative database searches, are useful for functional inferences. They improve sensitivity of pathway recovery when applied to a broad range of the known pathways, and they may also suggest new components of the known pathways, help define the composition of poorly studied pathways, and identify molecular correlates of specific phenotypic traits. At the same time, the specificity of probabilistic searches varies widely. This may be attributed in part to the shortcomings of the model, or to the low resolution intrinsic to the data. Indeed, even though Psi-square has been inspired by algorithms for analysis of molecular sequences, those approaches benefit from multi-state and multi-parameter sequence models, whereas phyletic vector models have only two character states and, in the current implementation, only one implicit transition probability. On the other hand, there is a clear distinction in the specificity of detection of different pathways: a large group of intermediary metabolic enzymes, centered on TCA and amino acid biosynthesis and linked to nucleotide biosynthesis, appears to form a giant component of the co-inheritance network, whereas many systems for interaction with the environment, such as systems of secretion and motility, as well as biosynthesis of certain coenzymes and utilization of secondary metabolites, are much easier delineated using our approach.

Glazko et al. [15] have shown that Psi-square may be usefully applied to many types of genome-wide datasets that can be represented in vector form, such as gene expression time series, genetic interactions, protein-protein interactions, and so on. An intrinsic limitation of many such datasets is that the number of coordinates of gene vectors may grow with time, but the number of genes (i.e., vectors themselves) cannot. A phyletic vector database, in contrast, is an example of a genomic dataset that will continue to grow in both dimensions: with the addition of novel genomes, the number of genes conserved in at least some of the genomes will also grow. Thus, just as Psi-square displays better sensitivity when applied to COG-06 vs. COG-04, we expect that phyletic pattern searches, when applied to even larger datasets in the future, will again show even greater sensitivity. It is our hope that they may also become more specific, especially if scoring functions employed by Psi-square and other methods are based on more sophisticated models of gene gain, loss and co-inheritance.

## Methods

### The data

#### Phyletic vectors of COGs

We used phyletic vectors from the most recent update of the NCBI COG database, as of June 2006 (Yuri I.Wolf, ftp://ftp.ncbi.nih.gov/pub/wolf/COGs/COG0508/). This dataset consists of 14,714 COGs from 110 complete genomes of archaea and bacteria and is referred to as COG-06 in this work. Each row of the data matrix represents the state of a specific COG in 110 genomes and each column represents the state of each of 14,714 COGs in a specific genome. In addition to standard NCBI COGs [1] which include genes from at least three lineages, we also included genes present in fewer than 3 organisms. This improves calculation of the background frequencies but does not affect the results otherwise (not shown).

The COG-06 dataset covers genomes from 16 archaea and 94 bacteria (the list of genomes is provided in Table S6). We also used an earlier release of the COG database [6], referred to as the COG-04 dataset, which includes genomes from 13 archaea and 50 bacteria (http://www.ncbi.nlm.nih.gov/COG/).

#### Phyletic vectors of phenotypes

The phenotype dataset was downloaded from the Genomes Online Database [28] on August 14, 2006. The data include descriptions of phenotypes, ecotype and related disease for a total of 2,125 genomes, of which 521 of are completely sequenced. We grouped phenotypes into 20 sets based on the symptoms of disease, ecotypes and phenotypic similarities (Table S3). Phyletic vectors for these 20 phenotypes for the 110 genomes that appear in the COG-06 dataset were constructed on the basis of presence or absence of the specific phenotype.

### Selection of appropriate distance measure and threshold for Psi-square similarity search

The performance of Psi-square depends on the choice of distance/similarity measure and several search parameters including *r*, the threshold admitting closely related vectors into the target set in the first step of the algorithm. In an earlier work [29], we have hypothesized that the statistical properties of distribution of pairwise distances between vectors, i.e., the values of the higher moment of distribution, can be used as guidance for selecting the distance measure for vector comparison. Using this criterion, we selected the correlation coefficient and Simpson similarity index as appropriate distance measures depending on the sparseness of the phyletic vectors in this work. For our datasets, it is difficult to use a fixed threshold *r* to admit closely related vectors into the target set for different query vectors, because some COGs are more closely related with correspondingly small distance, while other COGs are more distantly related. Therefore, choices of parameters in this work are based on preliminary testing and summary statistics from the distance distribution between the query vector and all vectors in the database. One the other hand, the value that this threshold *r* is set to is not critical since the results do not differ much as long as *r* is set in a reasonable range (data not shown).

### Sensitivity and specificity

In this work, sensitivity is defined as the percentage of COGs retrieved by a search that belong to the same pathway or functional system as the query. Specificity is defined as the ratio of true positives (the absolute number of retrieved COGs that are in the same pathway or functional system as the query) to all COGs retrieved by Psi-square.

## Supporting Information

Table S1 The list of 52 pathways and functional systems.
(0.02 MB XLS)

Table S2 Sensitivity comparison between clustering algorithm and Psi-square on COG04 and COG06 datasets.
(0.02 MB XLS)

Table S3 The list of complied phenotypic traits obtained from the Genomes OnLine Database (GOLD).
(0.01 MB XLS)

Table S4 Psi-square search results for cell motility phenotype.
(0.02 MB XLS)

Table S5 Psi-square search results for food poisoning phenotype.
(0.04 MB XLS)

Table S6 The list of genomes in the COG-06 dataset.
(0.03 MB XLS)

Table S7 X axis and Y axis labels in Figure 2.
(0.02 MB XLS)
